# 118 Outcomes of Amputation Following Electrical Burn Injuries: A Five-Year Follow-Up

**DOI:** 10.1093/jbcr/irad045.091

**Published:** 2023-05-15

**Authors:** Eunyeop Kim, Karen Kowalske, Kyra Jeanine Solis, Bingchun Wan

**Affiliations:** University of Texas Southwestern Medical School, Dallas, Texas; UT Southwestern Medical Center, Dallas, Dallas, Texas; UT Southwestern Medical Center, Dallas, Texas; UT Southwestern Medical Center, Dallas, Texas; University of Texas Southwestern Medical School, Dallas, Texas; UT Southwestern Medical Center, Dallas, Dallas, Texas; UT Southwestern Medical Center, Dallas, Texas; UT Southwestern Medical Center, Dallas, Texas; University of Texas Southwestern Medical School, Dallas, Texas; UT Southwestern Medical Center, Dallas, Dallas, Texas; UT Southwestern Medical Center, Dallas, Texas; UT Southwestern Medical Center, Dallas, Texas; University of Texas Southwestern Medical School, Dallas, Texas; UT Southwestern Medical Center, Dallas, Dallas, Texas; UT Southwestern Medical Center, Dallas, Texas; UT Southwestern Medical Center, Dallas, Texas

## Abstract

**Introduction:**

Studies have acknowledged the prevalence of amputation in patients with electrical burns; however, the exact probability of amputation as a result of electrical injuries and the effect of amputation on the long-term health remain unclear. The purpose of this study was to analyze electrical burn survivors with amputation using a large multi-center database and assess their corresponding long-term physical and mental health, compared with a non-electrical burn population with amputation.

**Methods:**

Retrospective reviews of burn patients from 1993 to 2021 were performed using the Burn Model System National Database, that yielded 408 patients with electrical etiology and 6341 with non-electrical etiology. The variables for analysis included the Veterans RAND 12-Item Health Survey and the Patient-Reported Outcomes Measurement Information System 29, collected at discharge, six months, 12 months, 24 months, and five years post-burn.

**Results:**

Electrical burn patients showed a higher amputation rate (30.3%) than non-electrical burn patients (6.6%) (p < 0.0001). Electrical burn patients with amputation showed significantly lower physical component scores (PCS=34.1) and physical function scores (PFS=37.1) at discharge than electrical burn patients without amputation (p < 0.05). No significant differences were found in mental component scores, anxiety, depression, sleep, or fatigue between patients regardless of burn type or amputation. Electrical burn patients experienced similar post-injury trends compared with non-electrical burn patients. Non-electrical burn survivors with amputation had the worst outcomes of all patient groups. The Hispanic population comprised the largest percentage of the electrical burn group (80%).

**Conclusions:**

This study shows that patients with amputation need extra attention to their physical impairment at discharge. Early rehabilitation in electrical burn patients with amputation should be emphasized. Non-electrical burn survivors with amputation need the most support from both physical and psychological perspectives. This study like many others supports that regardless of burn etiology, all burn survivors require psychological support at discharge. The study also accentuates the need for better electrical injury prevention and rehabilitation program for those who speak Spanish.

**Applicability of Research to Practice:**

More intensive and comprehensive interventions for electrical burn survivors with amputation in the earlier stages of rehabilitation can help ensure better long-term physical, psychological, and social outcomes. In contrast, rehabilitation plans for patients with non-electrical burns with amputation should concentrate more on the long-term management for up to a minimum of two years post injury, as these patients face significantly longer and severe difficulties than electrical burns patients with and without amputation.

